# The Role of Aquaporin 5 (AQP5) in Lung Adenocarcinoma: A Review Article

**DOI:** 10.3390/cells12030468

**Published:** 2023-01-31

**Authors:** Lukasz Jaskiewicz, Anna Romaszko-Wojtowicz, Anna Doboszynska, Agnieszka Skowronska

**Affiliations:** 1 Department of Human Physiology and Pathophysiology, School of Medicine, Collegium Medicum, University of Warmia and Mazury in Olsztyn, 10-082 Olsztyn, Poland; 2 Department of Pulmonology, School of Public Health, Collegium Medicum, University of Warmia and Mazury in Olsztyn, 10-719 Olsztyn, Poland

**Keywords:** aquaporin 5, AQP5, NSCLC, lung adenocarcinoma, cancer

## Abstract

Aquaporins (AQPs) are selective, transmembrane proteins, which are primarily responsible for the transport of water and small molecules. They have been demonstrated to play a key role in the development and progression of cancer. Lung adenocarcinoma is the most common primary lung cancer diagnosed in patients in Europe and the USA. The research done so far has provided firm evidence that some AQPs can be biomarkers for various diseases. The objective of this review article is to present a potential role of AQP5 in the development of lung adenocarcinoma. Original papers discussing the involvement of AQP5 in carcinogenesis and containing relevant clinical data were identified. In order to analyze the research material in accordance with PRISMA guidelines, a systematic search of the ScienceDirect, Web of Science, and Pubmed databases was conducted. Out of the total number of 199 papers identified, 14 original articles were subject to analysis. This article presents the pathophysiological role of AQP5 in the biology of lung adenocarcinoma as well as its prognostic value. The analysis substantiates the conclusion that the prognostic value of AQP5 in lung cancer requires further research. Another aim of this paper is to disseminate knowledge about AQPs among clinicians.

## 1. Introduction

Lung cancer is the leading cause of death worldwide. The majority of its cases (approximately 80%) are non-small cell lung cancer (NSCLC) subtypes [1]. High mortality is attributed to the fact that the diagnosis of cancer is usually made in the advanced stage of the disease. Compared to other neoplasms, the possibility of effective surgical treatment is limited, while other conventional therapies, chemo- and radiotherapy, do not produce satisfactory results. Thanks to newly developed diagnostic technologies (including the assessment of molecular mutations) and immunotherapy, the survival time of patients is observed to have been extended; however, the overall prognosis remains poor. The rate of carcinoma progression is defined by genetic changes in cancer cells as well as rearrangement of tumor microenvironment components [2]. It is, therefore, essential to conduct research on reliable biomarkers, which could be used for the assessment of prognosis and which might help to develop other/novel treatment options for patients with NSCLC.

Aquaporins (AQPs) are a family of small, tetrameric (up to 37.2 kDa/monomer) membrane proteins, selectively mediating the transport of water through cell membranes in response to osmotic gradients [3,4]. AQPs are expressed in many organs, including the brain, heart, skin, and kidneys. They are involved in transmembrane water permeability, with additional roles in skin hydration and fat metabolism, as well as cell migration and proliferation [5].

To date, 13 aquaporins have been identified in the human body, which fall into three classes in terms of their selectivity towards channeled substances. These are orthodox aquaporins (AQP0, 1, 2, 4–6, and 8), which are selectively permeable only to water molecules, aquaglyceroporins (AQP3, 7, 9, and 10), which also transport glycerol and urea, and superaquaporins (AQP11, 12). Thus far, the presence of four aquaporin isoforms has been determined in the lungs: AQP1 in endothelial cells, fibroblasts and mesothelium cells, AQP3 and 4 in the epithelium of the airways, and AQP5 in type I pneumocyte epithelium cells, submucosal glands, and airway epithelium cells [6].

Impaired expression of AQPs is linked to such pathological conditions as diabetes insipidus, obesity, Sjögren syndrome, or neoplastic diseases [7,8,9,10]. Increased expression of AQP genes in various types of tumors has been revealed, indicating their potential effect on the activity of neoplasms. AQPs play an important role in vascular permeability and tumor interstitial fluid pressure [11]. Expression of AQPs has been demonstrated in NSCLC, where these proteins participate in the progression of a neoplasm, contribute to the growth of a tumor [12,13], can be partly responsible for cancer metastases, and facilitate cell migration by increasing the permeability to water and by enhanced formation of cell projections [14]. In their research paper, Kang SK et al. discussed the involvement of AQP5 in the activation of the Ras, ERK, and Rb signaling pathway secondary to the activation of Ras/ERK [15], and the cited authors demonstrated that AQP5 overexpression induced extracellular growth signals, which were transduced through the Ras-MAPK pathway to the nucleus, in which cyclin/CDK complexes phosphorylated Rb and triggered the transcription of genes involved in cell proliferation. Moreover, it is claimed that AQP5 can promote migration of neoplastic cells also by affecting the secretion of mucins [16,17,18]. Mucins are associated with early post-operative metastases in NSCLC. High expression of AQP5 leads to a considerable increase in the expression of the mucin MUC5AC. Thus, the upregulation of this mucin can be linked to a tendency towards malignancy. Based on the analyzed research papers, it is interesting to note that despite numerous studies, precise mechanisms underlying the up- and down-regulation of the four isoforms of AQPs and their relationship with the progression and prognosis of lung cancer remain unclear. 

The aim of this paper is to present the potential role of AQP5 in the development of non-small cell lung cancer based on a review of research papers, and to promote the knowledge on AQPs among clinicians. We have chosen AQP5 because of its potential relationship with the activation of epidermal growth factor receptor (EGFR), extracellular receptor of kinase (ERK1/2), and p38 mitogen-activated protein kinase (p38 MAPK) in neoplastic cells [18]. This review article presents the current research in medical sciences dealing with the mechanisms of AQPs regulation and expression as well as their effect on lung cancer development.

## 2. Materials and Methods

### 2.1. Search Strategy to Identify Studies

**Figure 1:**
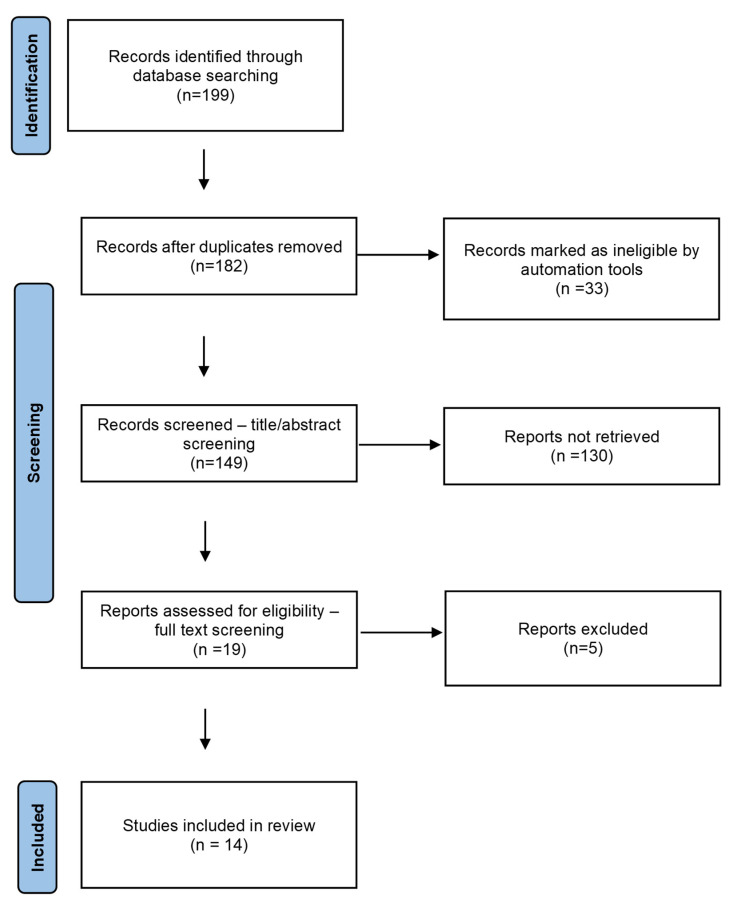
PRISMA protocol of search strategy and results [21].

The systematic review of literature was based on an a priori definition of inclusion and exclusion criteria, which allowed us to identify, review, and objectively select the references concerning the relationships of AQP5 with lung cancer [19]. The key words used were lung cancer, aquaporin, cancer, adenocarcinoma, and neoplasms. The following databases were searched using the above key words: ScienceDirect, Web of Science, and Pubmed. While searching the ScienceDirect database, an exclusion filter was applied to discard meta-analyses, reviews (including systematic reviews), and books, and the search was limited to publications in the domain of medical sciences. As regards the Web of Science database, the search was limited to publications in the fields of medicine, physiology, and pathophysiology. In Pubmed, the search filters used were the same as applied to the ScienceDirect database. The search was limited to papers in the English language. Detailed search results are presented in the diagram in Figure 1 [20]. The search was carried out in the first and second week of June 2022.

### 2.2. Selection of Research Papers and Inclusion Criteria

Rayyan software was used for further preselection of the papers [22]. In the first stage, duplicates were removed, and afterwards abstracts were analyzed to choose the papers dedicated to AQP5 and lung cancer. Titles and abstracts from the electronic search were evaluated independently by two reviewers. Disputes were resolved by consensus. In total, 182 articles were analyzed, of which 19 were selected for a further analysis of their full texts, and finally 14 articles from 12 journals were chosen for the review (Table 1). 

The papers were divided into two groups: laboratory preclinical studies, which were conducted solely on laboratory animals and/or cell lines, and clinical studies, where human material was used. A detailed division according to the subject fields is presented in Table 2.

## 3. Results

The analyzed papers were published between 2007 and 2021. Among the 14 articles subjected to our analysis, nine were dedicated exclusively to preclinical trials, four papers dealt with clinical trials, and one article concerned both preclinical and clinical trials. Most of the studies were carried out in Asia (*n* = 12) and the remaining ones were conducted in North America (*n* = 2).

### 3.1. Preclinical Trials

In the preclinical trials concerning AQP5, the role/participation of this aquaporin in signal transduction pathways associated with EGFR mutations has been demonstrated. EGFR is responsible for encoding a transmembrane tyrosine kinase with a ligand-binding extracellular domain and an intracellular component comprising a tyrosine kinase domain. The binding of epidermal growth factor ligands induces homo- and hetero-dimerization of the receptor with other members of the EGFR family and activation of a tyrosine kinase domain. Activating mutations in EGFR lead to the constitutive activation of tyrosine kinase and oncogenic transformation of lung epithelium cells in vitro. Transduction of signal is stimulated by EGFR proceeds through three signaling pathways: PI3K/AKT/mTOR, RAS/RAF/MEK/ERK MAPK, and JAK/STAT [36,37,38].

**Figure 2:**
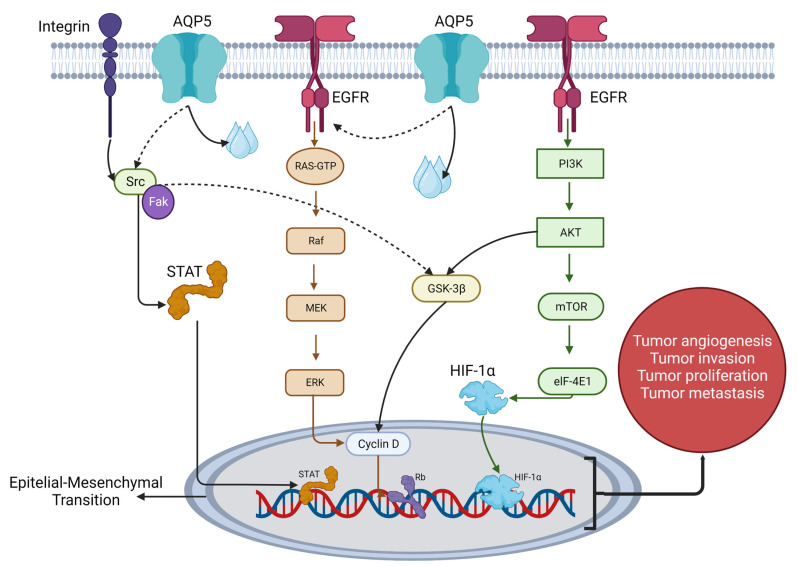
Signaling pathways associated with AQP5 in lung adenocarcinoma. AQP5 affects the activation of the extracellular kinase receptor (ERK1/2) pathway by activating the epidermal growth factor receptor (EGFR). In addition, AQP5 phosphorylated on Ser156 bind the SH3 domain of Src. In this way AQP5 participates in tumor invasion, metastasis, angiogenesis, and proliferation through a variety of complex signaling pathways. AQP5, aquaporin 5; EGFR, epidermal growth factor receptor; PI3K, Phosphoinositide 3-kinase; PDK1, 3-phosphoinositide-dependent protein kinase-1; AKT, protein kinase B; Raf, rapidly accelerated fibrosarcoma; MEK, mitogen-activated protein kinase; ERK, extracellular-signal-regulated kinase [39,40]. Created with BioRender.com.

A transgenic mouse model with inducible expression of the most common EGFR mutations demonstrated the development of many ADCs in lungs that were sensitive to small-molecule inhibition [30]. As regards AQP5, its effect on the RAS/RAF/MEK/ERK MAPK pathway has been documented. It was first reported in 2007, and subsequent studies confirmed the involvement of AQP5 in the modulation of this signal transduction pathway at its different stages [23] (Figure 2).

### 3.2. Clinical Trials

Studies on human tissues were initiated due to a potential role of AQP5 in progression of lung cancer and its involvement in EGFR signaling pathways. In 2008, AQP5 overexpression was proved to be associated with an elevated risk of the disease progression [25]. The papers published in the years after demonstrate that increased expression of AQP5 is connected with a greater incidence of metastases in the lymph nodes and a shorter remission time. Undoubtedly, when immunohistochemistry is applied for assessment of APQ5 expression, the type of used antibodies as well as the number of trials are important factors, and these may have a great impact on the results achieved by particular researchers. However, a paper published in 2021 demonstrated that a higher expression of AQP5 correlates with longer survival time (OS) and that high AQP5 expression is associated with a large number of infiltrating B lymphocytes and CD4^+^ lymphocytes, that is, TILs (tumor infiltrating lymphocytes) [35]. Hence, the influence of AQP5 on lung cancer prognosis calls for further research.

## 4. Discussion

Numerous studies have demonstrated that AQP5 can be an effective therapeutic target in the fight against cancer [41]. On the basis of the articles analyzed in our paper, it can be concluded that the role of AQP5 in the process of neoplasm formation has been a subject of thorough research. Such investigations have revealed, for example, that increased overexpression of this protein is significantly correlated with many cancers, including lung, prostate, and breast cancers [42]. High expression of AQP5 in cancers of the digestive system is linked to greater progression of the disease, resistance to chemotherapy, and worse prognosis. The latest study by He et al. (2017) showed that the lowering of the AQP5 level inhibited hepatocellular carcinoma metastases and epithelial-mesenchymal transition [43]. Many studies point to the potential usefulness of AQP5 as a biomarker of neoplasms. In clinical conditions, upregulation of AQP5 is a sign of poor prognosis in neoplastic diseases [29,44]. A paper published by Bera et al., in 2022 in Nature confirmed a participation of AP5 in the dissemination of breast cancer cells. The increase in the viscosity of the extracellular fluid increases the mechanical load by inducing the actin related protein, which affects the polarization of the Na^+^/H^+^ exchanger (NHE1) interacting with AQP5. Induction of membrane tension activates the TRPV channel, increasing the influx of calcium and migration of cancer cells [45].

Overexpression of genes of the four AQPs isoforms which play an important role in the growth of tumor and metastases has been demonstrated in studies on lung cancer [12,18]. Unquestionably, AQP5 has a considerable part in proliferation, migration, and angiogenesis in NSCLC [32,33]. Furthermore, the in vitro study conducted on a lung adenocarcinoma cell line by Chen et al. (2011) showed that AQP5 was involved in the migration of SPC-A1 by regulating the cell volume [28]. Changes in the shape and volume of cells induced by water permeability mediated by AQP5 may be an important factor in the migration of cells. It is thought that initially neoplastic cells are joined by lamellipodium, and then the matrix is dissolved and cell migration proceeds [46]. AQPs increase water permeability, thereby facilitating the formation of these projections and contributing to the increased rate of migration. Endothelial cells are also involved in cell migration, which explains their influence on angiogenesis [47]. Upregulation of AQP5 is associated with cell differentiation and plays a role in the invasion of neoplastic cells [18]. Moreover, early progression of disease in patients with NSCLC is related to the overexpression of AQP5 [25]. Reports on the overexpression of AQP5 in colorectal cancer and in pancreatic cancer were a starting point in the attempt to understand the biochemical mechanism causing a rise in the expression of AQP5 [44,48] In their in vitro studies with the use of the NSCLC cell lines H1299 and 16HBE, Elkhider et al. (2020) showed that downregulation of AQP5 inhibited angiogenesis by decreasing the expression of vascular endothelial growth factor (VEGF) [33]. They also pointed to the role of AQP5 in progression of adenocarcinoma. It was interesting to observe that higher expression of AQP5 was noted in adenocarcinoma than in squamous cell carcinoma (68.0% vs 28.6%) [33]. In a study conducted by Sung et al., the expression of AQP5 did not have a significant effect on the correlations of this aquaporin with the pathological and clinical results, although a statistically significant effect on DFS (disease-free survival) (*p* = 0.47) was demonstrated for the low expression of AQP5, which in turn indicates that AQP5 might have a prognostic value [1]. This is consistent with the in vivo study on knockout mice carried out by Zhang et al., who proved that AQP5 overexpression in lung adenocarcinoma cells promoted nodular progression. Moreover, in an in vitro study using cell lines, these authors showed a significant increase in PCNA and c-myc expression as well as a significant rise in the expression of MUC5AC and MUC5B in cells transfected with AQP5, which proves that this aquaporin has influence on regulating the EGFR/ERK/p38 MAPK signaling pathway [26]. An association of AQP5 with the Ras pathway was first reported in 2007, by Woo et al., in their study on the NIH3T3 cell line [23]. It was then demonstrated that cAMP had a biphasic effect on AQP5 response, and that the Ras pathway participated in AQP5 signaling pathways through the blocking of MEK pathways. AQP5 is also able to activate the Ras pathway through phosphorylation of the PKA consensus site of AQP5 in the D cytoplasmic loop. In 2008, the same team of researchers (Woo et al.) put forth the hypothesis that AQP5 overexpression could act as an oncoprotein both in vitro and in vivo, and this association may arise from the upregulation of cAMP, which was verified in laboratory conditions on lung cancer cell lines [25]. Further in vitro studies on cell lines enabled the researchers to confirm that AQP5 transfected cells showed a significant increase in the expression of PCNA and c-myc. Moreover, a significant increase in the expression of MUC5AC was noted, which attested to the effect on regulation of the EGFR/ERK/p38 MAPK pathway signals. It is known that the EGFR signaling pathway is a significant element in the onset and progression of many neoplastic diseases. Activation of EGFR by the binding of a specific ligand or gene mutation promotes proliferation and migration of neoplastic cells, thereby raising their invasive potential and inhibiting apoptotic signals. The EGFR/ERK signaling pathway plays a key role in lung cancer metastases [18]. It has been demonstrated that levels of AQP5 expression are directly connected with the activity of the EGFR/ERK signaling pathway, for example p-ERK additionally activates the hypoxia-inducible factor (HIF)-1α, which leads to degradation of AQP5 transcription inhibitors and intensification of AQP5 transcription activators [49]. Moreover, the detection of a connection between the EGFR pathway and lung adenocarcinoma, where *inter alia* overproduction of mucins occurs, was a starting point for the research undertaken by Zhang et al., on a potential influence of AQP5 on the expression of MUC5AC and MUC5B. It is worth noting that tumors with high AQP5 expression were also characterized by overproduction of mucins [26]. Additionally, this could be indirect evidence for the connection of the EGFR pathway with AQP5 expression and its influence on the invasive potential of lung adenocarcinoma. 

The NFAT5 pathway has a significant effect on the pathogenesis of NSCLC, melanoma, breast cancer, and colorectal cancer. It has been reported that a decrease in the expression of AQP5 leads to decreased proliferation and metastases. NFAT5 belongs to the NFAT protein family, which has a DNA binding domain that is structurally similar to the Rel NF-kB homology region [50]. It has been demonstrated that NFAT5 regulates the 70 heat shock protein, A urea transporter, tumor necrosis factor (TNF), AQP1, AQP2, and AQP4 [51]. Guo et al. proved that NFAT5 promoted proliferation and migration of cells through the regulation of AQP5 expression [30]. In the lung adenocarcinoma cells, the decreased expression of NFAT5 lowers the proliferation and migration of cells, which is accompanied by a considerable decrease in the expression of AQP5 [30]. Moreover, overexpression of NFAT5 promoted the proliferation and migration of lung adenocarcinoma cells, which coincided with a considerable increase in the expression of AQP5. Thus, decreasing the expression of AQP5 can delay the progression of lung cancer. 

It is possible that AQPs could be broadly used in oncological treatment. The elucidation of the role of AQP5 in the process of carcinogenesis of lung adenocarcinoma can result in using this protein as a prognostic biomarker, a solution that the up-to-date research results seem to support. Using molecular diagnostics, which enables identification of predictive biomarkers (genetic and epigenetic traits of the neoplasm), should allow oncologists to select the most appropriate treatment for a specific patient. Assuming that the number of cells displaying AQP5 expression has an adverse effect on the course of the disease or its prognosis, an immunohistochemical assay of sampled material for this parameter can provide more information about the progression of a neoplastic process. However, it needs to be added that data concerning the relationship between the presence of AQP5 protein and survival time are controversial. Most research reports have not demonstrated a strong relationship between AQP5 expression and survival time. It appears that further studies on a large number of samples are necessary. 

In our recent research report, Jaśkiewicz et al. (2022), we proved a possibility to determine AQP3 and AQP4 expression in lung adenocarcinoma samples collected during the diagnostic process of a neoplastic disease, during the following tests: bronchoscopy and transthoracic tumor biopsy [52]. In our opinion and according to literature, precise elucidation of the effect of AQPs on the biology of neoplasms requires further studies; nevertheless, it is possible that new antineoplastic therapies will be developed in the future that will take into account the inhibition of AQPs, including AQP5. 

When analyzing the results of the studies completed to this date, it appears that the exact molecular mechanism underlying the role of AQP5 in the formation of tumors has not been fully elucidated. The genetic relationship between AQP5 and any known type of neoplasm has not been completely identified [41]. Studies on AQP5 are relatively new. Inhibition of the expression of AQP5 in gastric carcinoma cells by acetazolamide has been described, leading to the decrease in the potential of invasion and proliferation of neoplastic cells [53]. Using heavy metal compounds might seem a promising solution, as they are effective inhibitors of AQPs. However, their toxicity profile as well as lack of specificity considerably limit their application [54].

For instance, the use of IgG anti-AQP4 antibodies is the first line treatment in patients with neuromyelitis optica (NMO) [55]. This way, the determination of the expression of AQPs alone may soon become as significant as the determination of EGFR/RAS/ALK mutations. Thus, AQPs can become potential predictors of the response to antineoplastic therapy [56].

## 5. Conclusions

This article presents a review of the literature discussing the role of AQP5 in pathophysiology of lung cancer, with a special focus on lung adenocarcinoma. In particular, it offers an insight into molecular mechanisms underlying the process of carcinogenesis. However, the relationships between AQP5 and NSCLC, breast cancer or colorectal adenocarcinoma have not been fully elucidated, and, therefore, further studies are needed in order to verify the role of AQP5 in vivo and its application in clinical practice.

## Figures and Tables

**Table 1:** List of papers selected for the systematic review.

No	Authors	Year	Title	Journal	References Data
1.	Janghee Woo, Juna Lee, Myoung Sook Kim, et al.	2008	The effect of aquaporin 5 overexpression on the Ras signaling pathway	*Biochem. Biophys. Res. Commun*.	[23]
2.	Janghee Woo, Juna Lee, Young Kwang Chae, et al.	2008	Overexpression of AQP5, a putative oncogene, promotes cell growth and transformation	*Cancer Lett.*	[24]
3.	Young Kwang Chae, Janghee Woo, Mi-Jung Kim, Sung Koo Kang, et al.	2008	Expression of aquaporin 5 (AQP5) promotes tumor invasion in human non small cell lung cancer	*PLoS One*	[25]
4.	Ziqiang Zhang, Zhihong Chen, Yuanlin Song, et al.	2010	Expression of aquaporin 5 increases proliferation and metastasis potential of lung cancer	*J. Pathol.*	[18]
5.	Zi-Qiang Zhang, Zhu-Xian Zhu, Chun-Xue Bai, et al.	2011	Aquaporin 5 expression increases mucin production in lung adenocarcinoma	*Oncol. Rep.*	[26]
6.	Yuichiro Machida, Yoshimichi Ueda, Miyako Shimasaki, et al.	2011	Relationship of aquaporin 1, 3, and 5 expression in lung cancer cells to cellular differentiation, invasive growth, and metastasis potential	*Hum. Pathol*	[27]
7.	Zhihong Chen, Ziqiang Zhang, Yutong Gu, et al.	2011	Impaired migration and cell volume regulation in aquaporin 5-deficient SPC-A1cells	*Respir. Physiol. Neurobiol.*	[28]
8.	Tianhe Song, Hong Yang, James Chung Man Ho, et al.	2015	Expression of aquaporin 5 in primary carcinoma and lymph node metastatic carcinoma of non-small cell lung cancer	*Oncol. Lett.*	[29]
9.	Kai Guo, Faguang Jin	2015	NFAT5 promotes proliferation and migration of lung adenocarcinoma cells in part through regulating AQP5 expression	*Biochem. Biophys. Res. Commun.*	[30]
10.	Young Min Jo, Tae In Park, Hwa Young Lee, et al.	2016	Prognostic Significance of Aquaporin 5 Expression in Non-small Cell Lung Cancer	*J. Pathol. Transl Med.*	[31]
11.	Lin Zhang, Jia Lu, Hongyan Zhou, et al.	2018	Silencing of aquaporin 5 inhibits the growth of A549 lung cancer cells in vitro and in vivo	*Int. J. Oncol.*	[32]
12.	Abdalkhalig Elkhider, Bing Wang, Xunli Ouyang, et al.	2020	Aquaporin 5 promotes tumor migration and angiogenesis in non-small cell lung cancer cell line H1299	*Oncol. Lett.*	[33]
13.	Yuko Takashi, Kazuo Tomita, Yoshikazu Kuwahara, et al.	2020	Mitochondrial dysfunction promotes aquaporin expression that controls hydrogen peroxide permeability and ferroptosis	*Free Radical Biology and Medicine*	[34]
14.	Guofu Lin, Luyang Chen, Lanlan Lin, et al.	2021	Comprehensive Analysis of Aquaporin Superfamily in Lung Adenocarcinoma	*Front. Mol. Biosci.*	[35]

**Table 2:** Role of aquaporins in biology of adenocarcinoma.

No	Source	Species	Type of the Research/Method	Function and Features	References Data
**Clinical research with the use of human tissues**					
1.	Relationship of aquaporin 1, 3, and 5 expression in lung cancer cells to cellular differentiation, invasive growth, and metastasis potential	Surgical specimens from patients with lung cancer	Immunohistochemical	AQP5 does not have prognostic implications	[27]
2.	Expression of aquaporin 5 in primary carcinoma and lymph node metastatic carcinoma of non small cell lung cancer	Human lung tissues	Immunohistochemical staging	Expression of AQP5 is related to worse survival rates of patients with NSCLC	[29]
3.	Prognostic Significance of Aquaporin 5 Expression in Non-small Cell Lung Cancer	Normal human lung tissues	Immunohistochemical staging	Expression of AQP5 is related to worse prognosis in NSCLC	[31]
4.	Comprehensive Analysis of Aquaporin Super family in Lung Adenocarcinoma	Human lung tissues	Immunohistochemical staging	Expression of AQP5 relates to longer survival of patients with adenocarcinoma	[35]
**Preclinical research with the use of cell lines**					
5.	The effect of aquaporin 5 overexpression on the Ras signaling pathway	Human cDNA from HEK 293	Immunohistochemical	AQP5 affects regulation of the RAS signaling pathway	[23]
6.	Overexpression of AQP5, a putative oncogene, promotes cell growth and transformation	Lung cancer cell lineA431	Immunohistochemical	AQP5 affects proliferation of cells	[24]
7.	Expression of aquaporin 5 increases proliferation and metastasis potential of lung cancer	Human pulmonary adenocarcinoma cell lines (SPC-A1 and PC-9)	Immunohistochemical	AQP5 affects regulation of signals on the EGFR/ERK/p38 MAPK pathway	[18]
8.	Aquaporin 5 expression increases mucin production in lung adenocarcinoma	Human pulmonary adenocarci-noma cell lines (ltep-A2 and pc-9)	Immunohistochemical	AQP 5 effects an increase in the production of mucins through more intensive expression of MUC5AC and MUC5B	[26]
9.	Impaired migration and cell volume regulation in aquaporin 5-deficient SPC-A1cells	SPC-A1, a human lung adenocarcinoma cell line	Tumor cell migration and invasion Lipofectamine 2000	Down-regulation of AQP5 leads to a decrease in the migration and invasion of cells	[28]
10.	NFAT5 promotes proliferation and migration of lung adenocarcinoma cells in part through regulating AQP5 expression	Human lung epithelial cells and human NSCLC cell lines, A549, H1975, H460, andH226 cells	Tumor cell migration and proliferation Lipofectamine 2000	Decreased expression of AQ5 leads to a decrease in proliferation and metastases	[30]
11	Silencing of aquaporin 5 inhibits the growth of A549 lung cancer cells in vitro and in vivo	The cell lines A549, H358, HCC827, and H1299	Immunohistochemical	Down-regulation of AQP5 inhibits the ERK signaling pathway	[32]
12.	Aquaporin 5 promotes tumor migration and angiogenesis in non-small cell lung cancer cell line H1299	NSCLC (H1299 and 16HBE) i HUVEC cells	Tumor cell migration and angiogenesis	Inhibition of AQP5 leads to a decrease in expression of VEGF, secondarily contributing to the inhibition of angiogenesis and metastasis	[33]
13.	Mitochondrial dysfunction promotes aquaporin expression that controls hydrogen peroxide permeability and ferroptosis	The HeLa and SAS human cancer cell lines	Immunohistochemical	AQP 5 contributes to ferroptosis of cells (increased AQP5 expression contributes to apoptosis of neoplastic cells)	[34]
**Research with the use of cell lines and human tissues**					
14.	Expression of aquaporin 5 (AQP5) promotes tumor invasion in human non-small cell lung cancer	Normal lung cell line BEAS-2B murine fibroblast cell lineNIH3T3	Immunohistochemical	AQP5 can play a key role in NSCLC progression by increased cell invasion NSCLC	[25]

## Data Availability

Data available in a publicly accessible repository.
